# Preliminary validation of the insomnia severity index in Danish outpatients with a medical condition

**DOI:** 10.1186/s41687-020-0182-6

**Published:** 2020-03-02

**Authors:** Karin Brochstedt Dieperink, Caroline Matilde Elnegaard, Bodil Winther, Anna Lohman, Ida Zerlang, Sören Möller, Graziella Zangger

**Affiliations:** 1 Department of Oncology, Odense University Hospital, Odense, Denmark; 2 REHPA, the Danish Knowledge Centre for Rehabilitation and Palliative Care, University of Southern Denmark and Odense University Hospital, Odense, Denmark; 3 Academy of Geriatric Cancer Research, AgeCare, Odense University Hospital, Southern Boulevard 29, DK-5000 Odense C, Denmark; 4 Department of Oncology, Lillebaelt Hospital, Vejle, Denmark; 5 Department of Oncology, Zealand University Hospital, Naestved, Denmark; 6 Department of Oncology, Regional Hospital West Jutland, Herning, Denmark; 7 OPEN - Open Patient data Explorative Network Odense University Hospital, Odense, Denmark; 8 Department of Clinical Research, University of Southern Denmark, Odense, Denmark

**Keywords:** Sleep disorders, Insomnia, Reliability, Validity, Patient reported outcome measures

## Abstract

**Purpose:**

Insomnia is a frequent sleeping disorder in the general and clinical population. With an increasing proportion of health care services being provided as outpatient care, a short, valid and reliable tool is needed to identify insomnia in medical patients under outpatient care in Denmark. The Insomnia Severity Index (ISI) could be the needed tool if found valid and reliable. Hence, the aim of this study is to evaluate elements of the psychometric properties of the Danish version of ISI (ISI-DK).

**Methods:**

Outpatients from three hospital wards and one rehabilitation center were asked to complete the ISI-DK twice, 2 weeks apart. Internal consistency, discriminative validity, test-retest reliability, and measurement error was assessed.

**Results:**

The ISI-DK was completed by 249 (79.0%) participants the first time, and 163 (65.5%) the second time. Respondents had a mean age of 58.2 years (SD 13.5) and 63.5% were women. All but one of the discriminative hypotheses was accepted. Internal consistency was high in the global scale at 0.90 and good with Cronbach’s alpha at 0.75–0.88 in the proposed subscales. The test-retest reliability was good, as the intraclass correlation was 0.90 (95% CI: 0.87; 0.93). Ceiling and floor effects were low < 4.4%. Standard error of measurement was 2.52 and smallest detectable change 6.99.

**Conclusion:**

This preliminary assessment showed encouraging results supporting the ISI-DK as a valid and reliable tool for screening insomnia severity in Danish outpatients with a medical condition, but further assessments are needed.

## Introduction

Insomnia, characterized by having difficulties initiating or maintaining sleep, early awakening and/or poor sleep, is one of the most common sleep disorders with prevalence rates in general populations between 10 and 20% [1, 2]. With a major health impact, insomnia has been shown to diminish quality of life [3] and increase the risk of morbidity and mortality [4]. Insomnia is often concurrent with other medical conditions [5] and research has shown that almost 10% of the patients seen in the primary care setting suffer from chronic insomnia [3]. To prevent chronic insomnia, it is important to diagnose symptoms and initiate treatment of the underlying causes to prevent further morbidity. According to the European Insomnia Guideline, the diagnostic procedure for insomnia should include a clinical interview with sleep history, sleep diaries, and a physical examination [2]. However, insomnia screening is a useful and cost-effective method to separate patients with minor or temporary symptoms from patients with severe symptoms before referring patients to further diagnostics. Thus, insomnia screening is dependent on validated tools with a low administrative burden. The use of patient reported outcomes (PRO) has therefore become increasingly important in assessing the impact of insomnia and its treatment of health status and daily functioning [2].

The Insomnia Severity Index (ISI), developed by Morin [6], is currently one of the most used PRO insomnia questionnaires. The ISI is a brief seven item self-rated instrument, increasingly used to assess insomnia based on criteria from the International Classification of Sleep Disorders. The ISI has been translated into multiple languages, validated in 12 countries and as a web-based measurement [7–18]. In comparison to other PRO sleep measures, the ISI has diagnostic properties [19], and can be completed in a few minutes [8], diminishing the response burden. Of the few insomnia-specific PRO instruments available, the ISI is designed to capture patient-perceived insomnia severity and impact on daytime functioning.

A short, valid, and reliable tool is needed to identify insomnia in patients under outpatient care, as a growing proportion of health care services in Europe, and especially in Denmark, are provided as outpatient care [20, 21]. The ISI has the potential to become this valuable tool. It has already been translated into more than 50 languages, including Danish, by the Mapi Research Trust, the official distributor of the ISI [22]. However, although the translation process was professionally controlled, according to the primary author of the ISI Charles M. Morin, no study has investigated the measurement properties of the Danish version of the ISI and with his agreement this study was executed.

## Aim

The aim was to examine elements of construct-related validity and reliability of the Danish version of the Insomnia Severity Index (ISI-DK) in a population of outpatients with a medical condition in Denmark.

## Methods

### Design

This study was designed as a longitudinal test-retest study including psychometric testing to explore elements of validity and reliability [23]. Validity is perceived as the degree to which the questionnaire actually measures the concept that it is intended to measure, whereas reliability refers to the accuracy and the absence of measurement error of the instrument. The Consensus-based Standards of the Selection of Health Measurement Instruments (COSMIN) was used in the psychometric evaluation [23].

### Participants

With no solid scientific recommendation for the sample size needed for validation studies [24], the aim was to encompass the standard provided by COSMIN [23], which states that a sample of ≥100 is needed to attain a high quality psychometric assessment. To account for non-responders and with the available patients within the study timeframe, 315 participants were included. The sample was collected as a convenience sample from four sites; three Danish hospitals, aiming to recruit *n* = 50 outpatients at each site, and *n* = 165 patients recruited from those attending a residential rehabilitation stay at REHPA, the Danish Knowledge Centre for Rehabilitation and Palliative Care (www.rehpa.dk).

Hospital participants were included from three separate outpatient wards (medical, surgical and oncology). The REHPA participants were obtained from a group of patients with mixed cancer-diagnoses.

Exclusion criteria were age < 18 years, inability to understand written and spoken Danish or a cognitive impairment resulting in an inability to understand study instructions. Enrolment eligibility was evaluated by the researchers’ who approached potential participants.

The first questionnaire was filled out in person by the hospital outpatients, but e-mailed to the REHPA participants. The second questionnaire was sent by e-mail to all participants, 2 weeks after the first questionnaire was received, aiming for a two-week interval between test and retest. Reminder e-mails were sent to non-responders once or twice depending on replies, with 5 days in between reminders.

### Measurements

The first questionnaire consisted of demographic questions, the EuroQoL EQ-5D-5 L [25] (EQ-5D) and the ISI-DK. The second questionnaire consisted only of the ISI-DK.

#### EQ-5D

Health state was investigated by the generic, standardized and well validated EQ-5D. EQ-5D rates impairment level across five dimensions (mobility, self-care, usual activities, pain/discomfort and anxiety/depression), in 5 items scored on a 5-point Likert-type scale (no problems to extreme problems). Global health was assessed using the 0–100 points EQ-5D vertical, visual analogue scale (EQ VAS), where higher scores indicate better health.

#### The Insomnia Severity Index

The ISI includes seven items evaluating perceived severity of insomnia with a 2-week recall (for original items, please see Bastien et al. [8]). The respondent rates the severity of difficulties falling asleep; maintaining sleep; early morning awakenings; the degree of satisfaction with current sleep; level of interference of sleep difficulties with daytime functioning; the degree to which others notice the deterioration of functioning related to the sleep problem; and the level of worry or distress caused by sleep difficulties. A 5-point Likert-type scale (0–4) is used for scoring the items according to the perceived degree of severity. The total score is summed from the seven items ranging from zero to 28. A higher score indicates greater severity of insomnia. Cut-offs suggested by Bastien et al. [8] are used.

### Statistics

Data were entered into a SurveyXact database with the questionnaire set up so that missing data was not possible, ensuring a dataset without any missing items. To ensure high data quality, random checks were completed. IBM SPSS Statistics for Windows, Version 24.0. Armonk, NY: IBM Corp, was used for statistical analysis.

Descriptive statistics were used for demographics. Data are presented as frequencies, percentages, means, and standard deviations (SD); *p* < 0.05 was considered statistically significant. EQ-5D level of problem was dichotomized and shown as percentage of any problem (slight to extreme). Further, EQ-5D was converted into a single index value (EQ-index) ranging from 0 to 1 (1 = full health), using the available EuroQoL calculator [25]. ISI-DK floor and ceiling effects (i.e. the percentage of the lowest (0) and highest (28) scores, respectively), were evaluated and seen as troublesome if > 15%.

### Confirmatory factor analysis

Structure was examined by a confirmatory factor analysis (CFA) based on the maximum likelihood method. Before conducting the CFA, data suitability was tested by the Kaiser-Meyer-Olkin (KMO) test with a limit of > 0.60 and by Bartlett’s test of sphericity with a significance limit of *p* ≤ 0.05 [26]. The original article on ISI did not present a factor analysis, so the CFA was compared with previously tested models, with one [17, 27], two [28], and three [10, 13, 29] factors. The goodness of fit of each model was assessed by the fit indices; Root Mean Square Error of Approximation (RMSEA) value < 0.08, and Comparative Fit Index (CFI) > 0.90, Tucker–Lewis fit Index (TLI) > 0.95, and Standardized Root Mean Square Residual (SRMR value < 0.08 [30]. Further, lower values of Bayesian Information Criterion (BIC) and Akaike’s Information Criterion (AIC) was considered when finding the best fitted model. The proposed models are compared to model 1 by likelihood ratio test, to test if including subscales significantly improves the model fit of the structural equation modelling. The CFA was analysed using Stata statistical software version 16 (StataCorp. 2019. Stata Statistical Software: Release 16. College Station, TX: StataCorp LLC).

### Construct-related validity

Discriminative validity was measured by testing a priori hypotheses about subgroups expected to significantly differ in mean ISI-DK scores i.e. the known-group validation method. Five hypotheses were formulated by the author group primarily based on papers included in the review by Winkelman [31]. Our hypotheses were a priori formulated as follows:

- *Gender:* Female responders have significantly higher ISI-DK mean scores compared to male responders, as Roth et al. in the America Insomnia Survey, found that female gender was a predictor of insomnia [31, 32].
- *Age*: Responders ≥70 years old have significantly lower ISI-DK mean scores compared to younger responders, as Roth et al. points out that insomnia diagnoses are not more frequent in the elderly, because the effects of sleeplessness on daytime functioning appear to be less dramatic [31, 32]. The age limit of 70 years is selected as most Danes would be retired at that time point.
- *EQ VAS*: Responders with EQ VAS score < 83.7 have significantly higher ISI-DK mean scores compared to responders with higher EQ VAS scores, as several studies conclude that insomnia negatively affects quality of life [3, 31]. The EQ VAS cut-off was set using the Danish population norm total [33]
- *EQ-5D anxiety/depression:* Responders with any EQ-5D anxiety/depression problem have significantly higher ISI-DK mean scores compared to responders with no problem, as found by Ford et al. [31, 34], but also recently by Geoffroy et al. [35].
- *EQ-5D pain/discomfort:* Responders with any EQ-5D pain/discomfort problem have significantly higher ISI-DK mean scores compared to responders with no problem, as Morin et al. points out that insomnia often co-occurs with pain [36].

Groups were compared by t-tests for subgroups of *n* ≥ 50. Cohen’s d was calculated to estimate the size of the possible difference in ISI-DK mean score between each of the sub-groups and accordingly interpreted as small (0.2), medium (0.5) and large (0.8) [37, 38].

### Reliability

Internal consistency was assessed by Cronbach’s alpha (α) and item-total correlations. Cronbach’s α was tested in the overall scale and in the factors presented by the CFA. As an investigation of stability over time, test-retest reliability was assessed by intraclass correlation coefficient (ICC) by a two-way random effect model for absolute agreement. Both reliability measures were considered acceptable at a level of > 0.70 [39]. Reliability coefficients > 0.70 reflect that the questionnaire can be used for group comparisons (e.g. research purposes), where questionnaires that are used for individual assessment (e.g. clinical purposes) should be above the limit of 0.90 [40]. As parameters of measurement error i.e. systematic and random error of a patient’s score that is not attributed to true changes in the construct to be measured, standard error of measurement (SEM), smallest detectable change (SDC), and limits of agreement (LoA) were calculated [39]. Measurement error is expressed in the same units as the ISI-DK i.e. 0–28. The mean difference (meandiff) between the two tests was calculated by a paired-samples *t*-test. SEM was calculated by $\sqrt{mean} squareerror$ from ANOVA. The SDC refers to the minimal detectable change in scores on the ISI-DK, which indicates a real change beyond measurement error. SDC is calculated by 1.96 ×  √ 2 × *SEM*. LoA is calculated by *meandiff* ± 1.96*SD*. The LoA is shown in a Bland-Altman plot which displays the 95% range of changes in scores between the test-retest. There is, to our knowledge, no criterion of a good or acceptable level of measurement error, so a clinical judgement of the constituent elements was made.

## Results

### Participants

In total, 315 patients were asked to participate and *n* = 249 agreed and completed the first questionnaire (response rate 79.0%). The respondents had a mean age of 58.2 years (SD 13.5) and 63.5% were women. Most respondents had cancer *n* = 165 (66.3%) and *n* = 34 (13.7%) used sleep medication, Table 1. A total of *n* = 163 returned the second questionnaire (response rate 65.5%) and were included in the test-retest.

**Table 1:** Data on demographics, Insomnia Severity Index and EQ-5D (*n* = 249)

Age, mean [SD] (range)	58.2 [13.5] (21–90)
Gender, *n* [%]	
Male	91 [36.5]
Female	158 [63.5]
Education, *n* [%]	
Less than 10 years	38 [15.3]
Youth education programme	9 [3.6]
Medium long education	150 [60.3]
Long education	21 [8.4]
Other (vocational education, etc.)	31 [12.4]
Employment, *n* [%]	
Employed	97 [39.0]
Unemployed	32 [12.9]
Pension	91 [36.5]
Other (sick leave, student, part time, etc.)	29 [11.6]
Living situation, *n* [%]	
Living alone	64 [25.7]
Living with spouse/partner and/or children	185 [74.3]
Participants at sights, *n* [%]	
Surgical ward (Naestved)	49 [19.7]
Medical ward (Vejle)	45 [18.1]
Oncology ward (Herning)	50 [20.1]
Rehabilitation centre (REHPA)	105 [42.1]
Medical condition, n [%] (multiple answers were possible)	
Heart disease	11 [4.4]
Cancer	165 [66.3]
Lung disease	3 [1.2]
Other (diabetes, sclerosis, arthritis, etc.)	56 [22.5]
Multiple diseases (two or more of the above)	14 [5.6]
Currently in treatment, *n* [%]	158 [63.5]
Uses sleep medication, *n* [%]	34 [13.7]
Insomnia Severity Index scores, *n* [%]	
0–7 = absence of insomnia	90 [36.1]
8–14 = sub-threshold insomnia	90 [36.1]
15–21 = moderate insomnia	63 [25.4]
22–28 = severe insomnia	6 [2.4]
EQ-5D-5 L, *n* [%]	
Mobility	
No problem	165 [66.3]
Any problem	84 [33.7]
Self-care	
No problem	216 [86.8]
Any problem	33 [13.2]
Usual activities	
No problem	93 [37.4]
Any problem	156 [62.6]
Pain/discomfort	
No problem	64 [25.7]
Any problem	185 [74.3]
Anxiety/depression	
No problem	132 [53.0]
Any problem	117 [47.0]

*SD* Standard deviation

### Health state

The results of EQ-5D levels are shown in Table 1. The percentage of any reported problems on each dimension of EQ-5D was highest within pain/discomfort with 74.3% and lowest in self-care (13.2%). The EQ-index score was 0.75 (SD 0.16) and the EQ VAS mean score was 68.2 (SD 20.9).

### The ISI-DK

None of the respondents had missing items in the ISI-DK. The mean score of the ISI-DK was 10.24 (SD 6.05) in the first test. Using the insomnia cut-offs 25.4% had moderate insomnia and 2.4% had severe insomnia, Table 1.

For retest respondents, the ISI-DK mean scores were 10.99 (SD 5.80) the first time and 10.95 (SD 5.94) the second time. A paired t-test showed no statistically significant difference between these two means (*p* = 0.86). Item 4 (Sleep satisfaction) had the highest mean (2.08, SD 1.13) and item 6 (Noticeability) the lowest (0.95, SD 0.93), Table 2.

**Table 2:** Fit indices of confirmatory factor analyses in different Insomnia Severity Index factor models (*n* = 249)

Model	Chi^2^	*p*-value	RMSEA	CFI	TLI	SRMR	AIC	BIC	*p*-value^*^
Threshold for fit indices	None	> 0.05	< 0.08	≥0.90	≥0.95	< 0.08	Lowest value	Lowest value	< 0.05
Model 1. *One factor: All 7 items*	87.030	< 0.001	0.145	**0.931**	0.896	**0.048**	4285.162	4359.028	ref
Model 2. *Two factors:* *Subscale 1: 1, 2, 3, 4* *Subscale 2: 5, 6, 7*	40.577	< 0.001	0.092	**0.974**	**0.958**	**0.033**	4240.709	**4318.093**	1.0
Model 3. *Two factors:* *Subscale 1: 1, 2, 3* *Subscale 2: 4, 5, 6, 7*	67.746	< 0.001	0.130	**0.948**	0.916	**0.042**	4267.877	4345.261	1.0
Model 4. *Three factors:* *Subscale 1: 1, 2, 3* *Subscale 2: 1, 4, 7* *Subscale 3: 5, 6, 7*	26.497	0.002	0.089	**0.983**	**0.961**	**0.025**	**4234.629**	4326.083	1.0

Bold text for the best fitted value for the indices

*RMSEA* Root Mean Square Error of Approximation, *CFI* Comparative Fit Index, *TLI* Tucker–Lewis fit Index, *SRMR* Standardized Root Mean Square Residual, *AIC* Akaike’s Information Criterion, *BIC* Bayesian Information Criterion

^*^Likelihood ratio test

The ISI-DK had low floor and ceiling effects of 4.4% (*n* = 11) and 0%, respectively.

### Confirmatory factor analysis

**Fig. 1:**
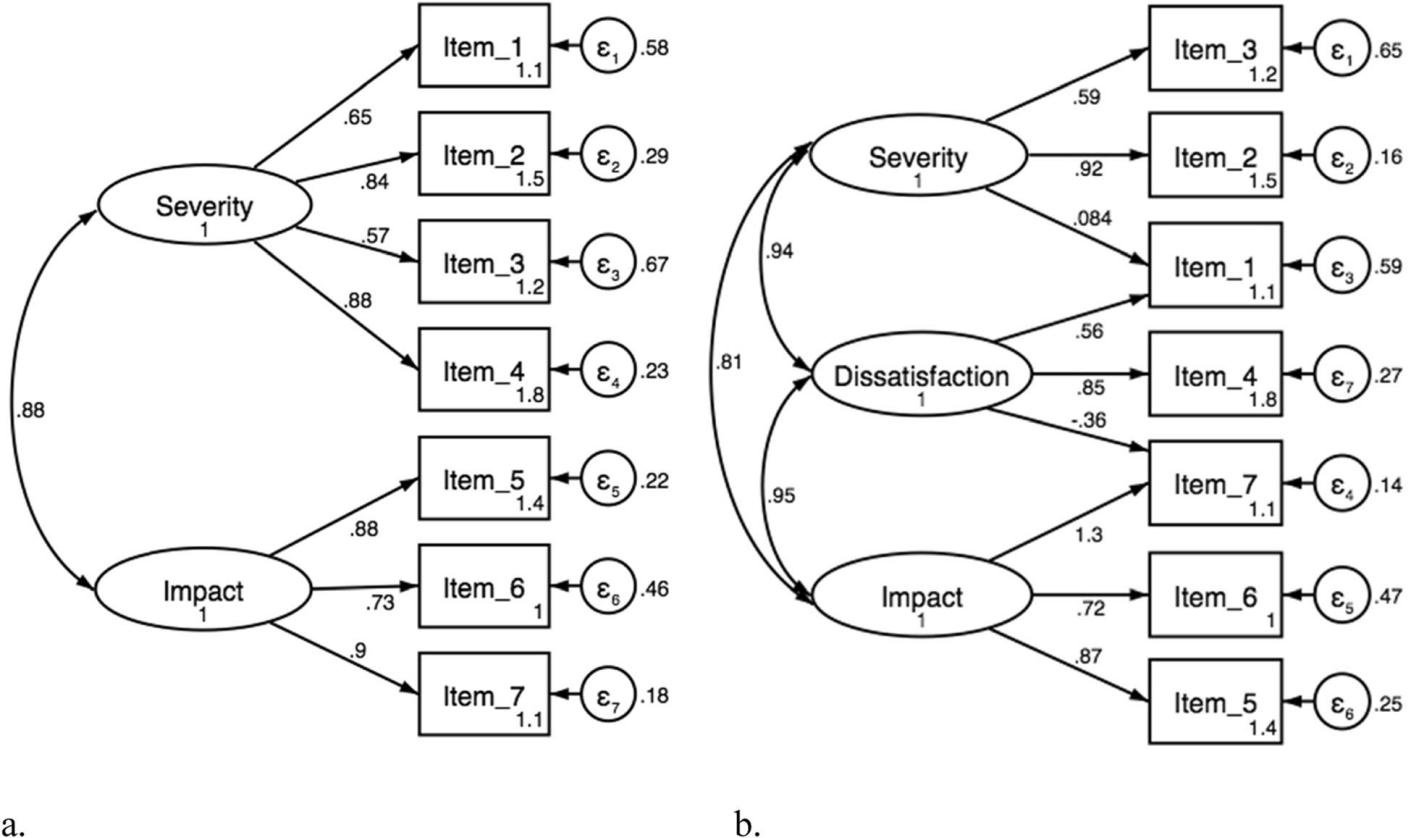
Showing the two (**a**., model 2) and three (**b**., model 4) factor models of the Insomnia Severity Index Danish version with estimated factor loadings, correlation between the factors, and the residual standard errors

Data was adequate for CFA with a KMO of 0.88 and a Bartlett’s test of *p* < 0.001. Results of the CFA are shown in Table 2. None of the models had a perfect fit, but both model 2 and 4 showed to fit more of the indices than the other two models, see Fig. 1. Judging by the AIC model 4 is the better fitted model, while BIC pointed to model 2. Testing if models 2, 3 and 4 nested in model 1 significantly improved the fit, no significant improvements were detected indicating the subscales were not improving the total fit significantly.

### Construct-related validity

Of the five discriminative hypotheses four were accepted (*p* < 0.001), Table 3.

**Table 3:** Discriminative validity of the Insomnia Severity Index Danish version (ISI-DK)

Hypotheses	Mean ISI-DK score (SD)
Gender: Female responders have significantly higher ISI-DK mean scores compared to male responders	
Male, *n* = 91	9.36 (6.03)
Female, *n* = 158	10.74 (6.02)
Effect size (Cohen’s *d)*	0.23
*p*-value	0.083
Confirmed hypothesis	No
Age: Responders ≥ 70 years old have significantly lower ISI-DK mean scores compared to younger responders	
≥ 70, *n* = 60	8.85 (6.55)
≤ 69, *n* = 188	10.65 (5.83)
Effect size (Cohen’s *d)*	0.40
*p*-value	0.044
Confirmed hypothesis	Yes
EQ VAS: Responders with EQ VAS score < 83.7 have significantly higher ISI-DK mean scores compared to responders with higher EQ VAS scores	
VAS score ≤ 83.6, *n* = 189	11.21 (6.00)
VAS score ≥ 83.7, *n* = 60	7.18 (5.13)
Effect size (Cohen’s *d)*	0.69
*p*-value	< 0.001
Confirmed hypothesis	Yes
EQ-5D anxiety/depression: Responders with any EQ-5D anxiety/depression problem have significantly higher ISI-DK mean scores compared to responders with no problem	
Anxiety/depression no problems, *n* = 132	8.23 (5.66)
Anxiety/depression problems, *n* = 117	12.50 (5.68)
Effect size (Cohen’s *d)*	0.75
*p*-value	< 0.001
Confirmed hypothesis	Yes
EQ-5D pain/discomfort: Responders with any EQ-5D pain/discomfort problem have significantly higher ISI-DK mean scores compared to responders with no problem	
Pain/discomfort no problems, *n* = 64	7.44 (5.28)
Pain/discomfort problems, *n* = 185	11.21 (6.01)
Effect size (Cohen’s *d)*	0.65
*p*-value	< 0.001
Confirmed hypothesis	Yes

Cut-off for effect size: 0.2 (small), 0.5 (medium) and 0.8 (large)

### Reliability

Cronbach’s α was very good (0.90) in the global scale supported by a high item-total correlation interval between 0.52–0.80, with a mean value of 0.71 (SD 0.11). Item 3 (Early awakening) had the lowest item-total correlation (0.52), but deleting the item would only increase Cronbach’s α slightly to 0.91. Cronbach’s α was also good in the factors found by the CFA, with values between 0.75–0.88. The retest had a Cronbach’s α of 0.91 with item-total correlation between 0.56–0.83 (mean 0.73, SD 0.11). Please see Table 4 for parameters of Reliability.

**Table 4:** Reliability, floor, ceiling effect, and measurement error of the Insomnia Severity Index Danish version (*n* = 163)

Floor effects, n [%]	11 [4.4]
Ceiling effects, n [%]	0 [0.0]
Reliability	
Internal consistency, Cronbach’s alpha [inter-item correlation range]	
Global scale (all 7 scale items)	0.90 [0.52–0.80]
Model 2	
Severity subscale (item 1, 2, 3, 4)	0.83 [0.74–0.88]
Impact subscale (item 5, 6, 7)	0.88 [0.85–0.92]
Model 4	
Severity subscale (item 1, 2, 3)	0.75 [0.38–0.55]
Dissatisfaction subscale (item 1, 4, 7)	0.81 [0.50–.072]
Impact subscale (item 5, 6, 7)	0.88 [0.63–0.79]
Test-retest reliability	
ICC [95%CI]	0.90 [0.87–0.93]
Measurement error	
SEM ($\sqrt{mean} square(ANOVA)$)	2.52
SDC (1.96 × √ 2 × *SEM*)	6.99
LoA (mean difference between test and retest) [95% limits]	0.05 [−6.92–7.02]

*ICC* Intraclass correlation, *CI* confidence interval, *SEM* standard error of measurement, *SDC* smallest detectable change, *LoA* limits of agreement

**Fig. 2:**
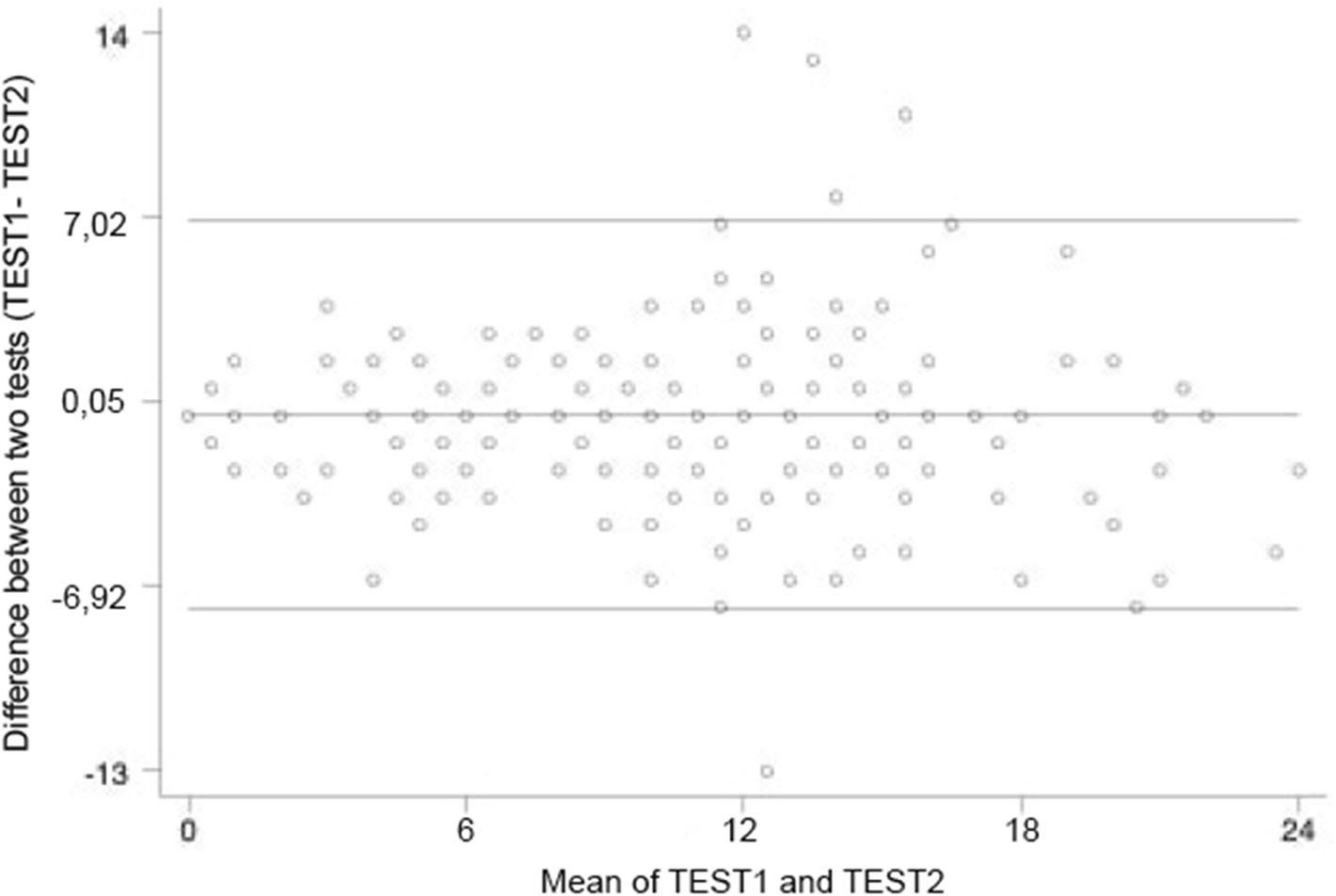
Bland-Altman plot of test-retest reliability of the Insomnia Severity Index Danish version

The test-retest mean interval was 17.1 days (SD 3.82, range 14–30 days). The test-retest reliability was found to be very good with an ICC value of 0.90 with a 95% confidence interval from 0.87 to 0.93, Table 4. SEM was found to be 2.52, and SDC was 6.99, Table 4. LoA is shown in the Bland-Altman plot, Fig. 2.

## Discussion

Our aim was to investigate elements of validity and reliability of the ISI Danish version and our main results support the questionnaire as a valid and reliable screening tool.

### Confirmatory factor analysis

The CFA results was unclear, with most fit indices pointing towards model 2 (2 factors) and 4 (3 factors) having the better fit. Previous results regarding the structure of the ISI have varied, showing different model structures within the seven items [8]. A three factor model was first presented in 2001 by Bastian et al. [8] who found a three factor division of the items, using a principal component analysis (PFA). Three factors was also found by Fernandez-Mendoza et al. [13], Chen et al. [29], and Castronovo et al. [10] by a CFA method. A two factor division of the items was found by Sadeghniiat-Haghighi et al. 2014 [15] using a PFA method and by Otte et al. 2019 [28] using a CFA. The tested three-factor model could be criticized as it has two items that cross load, which then makes for a less robust factor with only one unique item. This may be due to redundancy within the scale items and Dragioti E et al. 2015 [17] found that a scale consisting of only four ISI items was valid and reliable to assess insomnia.

Information criteria pointed to models 2 and 4 as best fitting data, but the general preference for more parsimonious models can be taken as an argument for preferring model 2, as the structure with only two factors and no overlap between subscales results in a simpler, and more interpretable model. The difference in structural validity could, to some extent, be caused by the wide variety of statistical models used, but also be due to the original construction of the scale, as one cohesive scale not constructed with a factor analysis [8]. Further, our sample may be too heterogeneous, and this could affect the results. However, the ISI is to our knowledge, clinically used as a one-dimensional scale offering only a single score for insomnia. A further division of the scale items into another model, did therefore not seem relevant. Testing if the nested models were significantly improving the fit, the tests for models with sub-scales (e.g. model 2, 3 and 4) were extremely non-significant, supporting the one factor model. However, a one factor model could be criticized, as the ISI items have a natural segregation, dividing items into insomnia severity and impact, and with multiple studies supporting three latent factors, the scale factor, scoring and interpretation may need to be revised. Nevertheless, the model fit in the current CFA was not convincing and the structural validity should be investigated further.

### Construct-related validity

One of the a priori discriminative hypotheses was not confirmed but it did show the expected tendency of women to have a higher ISI mean score than men. Assessing the Cohen’s *d,* the same result is found, that the gender hypotheses had a small effect. A previous study did find that the ISI could discriminate between subgroups within gender [41]. The results in the current study may be explained by a greater proportion of women within the study sample. The effect size of the other hypotheses was medium to large and these hypotheses may also be the more solid hypotheses with more evidence to underpin the hypotheses. This current Known-group analysis supports the ISI-DK as a discriminative tool, with the ability to distinguish between subgroups with insomnia.

### Reliability

Both internal consistency and the test-retest reproducibility were above the limit (0.70), thereby exceeding the criteria for group comparison. The global scale was satisfactory for comparisons on an individual level and importantly for clinical use (0.90) [42]. The Cronbach’s α results are comparable with previous studies that also found values above 0.70 [8, 11, 13, 43] and some also encompassed the level of 0.90 [41, 44]. A good internal consistency indicating homogeneity of the scale and inter-relatedness between items and as Cronbach’s α was not above 0.90 the items are most likely without redundancy [45]. The item-total correlations were also comparable with previous studies [18, 44, 46] and support that all items are relevant to the scale. One study also evaluated reproducibility assessed by ICC over a two-week period, and they found similar results with good stability (ICC > 0.84) [16]. The test-retest interval is one of the critical design challenges as it cannot be too short (i.e. risk of falsely high consensus between tests) or too long (i.e. a real change happens), both will affect the stability and thereby reliability [39]. In this study, the test-retest interval ranged widely around the pre-set 14 days, and this may have introduced bias, but a larger sample size was prioritized over a narrower test-retest interval.

### Measurement error

To our knowledge this study is the first to assess measurement error within the Danish version of the ISI. Measurement error is an indication of how accurate a score is [47], but there is no pre-set level of an acceptable amount of measurement error, and normative data are needed for a thorough interpretation. However, as a rule of thumb, a higher reliability is present when SEM and SDC are closer to zero [39]. SEM in our study was considered low (2.52) and indicates that the true sleep score for each participant is not far away from the obtained ISI-DK scores. The SDC is used in interpretation of how much scores must change beyond measurement error to be considered a true change and to distinguish this change from measurement error the SDC should be smaller than the minimal important change (MIC) [39]. Morin et al. [48] assessed MIC by evaluating sensitivity and specificity indices in receiver-operating curves using item response theory analyses, suggests that MIC > 7 is equal to moderately improved and > 9 corresponds to a major improvement. Thereby MIC exceeded SDC in this study and so it is possible to distinguish an important change from measurement error on the ISI scores. When comparing this to the ISI cut-offs presented by Bastien et al. [8] of 0–7, 8–14, 15–21, 22–28, the ISI score would need to change by an entire category to be considered a real change.

The LoA and mean difference indicates that the ISI-DK has good reliability, as it roughly measured the same scores over a two-week interval. The LoA range indicates that differences between the two tests in 95% of the cases differ with maximum − 6.92 to 7.02 which are comparable to the SDC result and to the MIC found by Morin et al. [48]. When related to the ISI score categories it also matches closely to a change of one category. Further, the LoA showed five participants with large differences between their test scores and who therefore were on the outside of the LoA. Unfortunately, the participants in the test-retest sample were not asked if they had experienced any life events that could have affected their sleep. Alternatively, this change could be a real change, demonstrating the reality that sleep patterns can change within a fortnight or participants could have experienced a respond shift and therefore have a different perception of their sleep problems at retest [39].

### Participants

Participants in this study demonstrated willingness towards completing the ISI-DK, as the response rate was rather high. As content validity was not assessed, it is not possible to know if the respondents felt a lack of certain insomnia related items, but as the questionnaire already has been applied and validated in many different patient groups the content validity was assumed. Some participants commented that three ISI-DK items are formulated in a way where it is assumed that the respondent has a sleeping problem, an issue that should be addressed if content validity is evaluated.

Prevalence rates for insomnia in cancer patients range from 25% to 60%, depending on definition, time of assessment and measurement tool used [49]. Thus, the incidence of insomnia in this study is well within what exists in both the general population and in cancer populations.

It is important to note that the ISI-DK, as with other screening instruments, requires that the respondents with insomnia have a healthcare professional interpret their insomnia problems and if necessary initiate an intervention [8].

### Strength and limitations

This study has both strengths and limitations. Strengthening the study was the design with multicenter recruiting sites resulting in a variety of diagnoses and cultural differences in a heterogeneous cohort. Also, a high level of accuracy in the data collection process was applied and the response rate was high, substantially strengthening the study. A limitation is that no other sleep assessment was administered concurrently limiting the evaluation of validity. Further, neither healthy controls nor sleep diaries were included. Both would have provided insight into whether changes in sleep pattern occurred and at what time points [48]. Strengthening the study was that participants were blinded to the performance of themselves and others [39] and participants with severe insomnia were contacted and instructed to consult their physician. Finally, a clear strength is that the COSMIN criteria were followed throughout the psychometric evaluation increasing the quality of the study.

## Conclusion

In conclusion, the preliminary psychometric assessment showed promising results and supports the ISI-DK as a valid and reliable tool for screening and assessing the severity of insomnia in Danish outpatients with a medical condition. Further research should assess response burden, compare the ISI-DK to other sleep measures to assess convergent and concurrent validity, evaluate responsiveness and reassess the model structure as well as the interpretation of the ISI score.

## Data Availability

The datasets used and analysed during the current study are available from the corresponding author on reasonable request.

## Abbreviations

AIC: Akaike’s Information Criterion
BIC: Bayesian Information Criterion
CFA: Confirmatory factor analysis
CFI: Comparative Fit Index
EQ-5D: European Quality of Life 5 dimensions
ICC: Intraclass correlation coefficient
ISI: Insomnia Severity Index
ISI-DK: Insomnia Severity Index - Danish version
KMO: Kaiser-Meyer-Olkin test
LoA: Limits of agreement
MIC: Minimal important change
PRO: Patient-reported outcome
RMSEA: Root Mean Square Error of Approximation
SD: Standard deviation
SDC: Smallest detectable change
SEM: Standard error of measurement
SRMR: Standardized Root Mean Square Residual
TFI: Tucker–Lewis fit Index
